# Chronic kidney disease of non-traditional origin in Mesoamerica: a disease primarily driven by occupational heat stress

**DOI:** 10.26633/RPSP.2020.15

**Published:** 2020-01-27

**Authors:** Catharina Wesseling, Jason Glaser, Julieta Rodríguez-Guzmán, Ilana Weiss, Rebekah Lucas, Sandra Peraza, Agnes Soares da Silva, Erik Hansson, Richard J. Johnson, Christer Hogstedt, David H. Wegman, Kristina Jakobsson

**Affiliations:** 1 La Isla Network La Isla Network Washington DC United States of America La Isla Network, Washington DC, United States of America.; 2 Pan-American Health Organization Pan-American Health Organization Washington DC United States of America Pan-American Health Organization, Washington DC, United States of America; 3 University of Birmingham University of Birmingham Birmingham United Kingdom University of Birmingham, Birmingham, United Kingdom; 4 University of El Salvador University of El Salvador San Salvador El Salvador University of El Salvador, San Salvador, El Salvador; 5 University of Gothenburg University of Gothenburg Gothenburg Sweden University of Gothenburg, Gothenburg, Sweden; 6 University of Colorado at Denver University of Colorado at Denver Aurora United States of America University of Colorado at Denver, Aurora, United States of America; 7 Karolinska Institutet Karolinska Institutet Stockholm Sweden Karolinska Institutet, Stockholm, Sweden; 8 University of Massachusetts Lowell University of Massachusetts Lowell Lowell United States of America University of Massachusetts Lowell, Lowell, United States of America

**Keywords:** Renal insufficiency, chronic, occupational health, disorder, heat stress, pesticides, metals, infections, Central America, Insuficiencia renal crónica, salud laboral, estrés por calor, plaguicidas, metales, infección, América Central, Insuficiência renal crônica, saúde do trabalhador, estresse térmico, praguicidas, metais, infecção, América Central

## Abstract

The death toll of the epidemic of chronic kidney disease of nontraditional origin (CKDnt) in Mesoamerica runs into the tens of thousands, affecting mostly young men. There is no consensus on the etiology. Anecdotal evidence from the 1990s pointed to work in sugarcane; pesticides and heat stress were suspected. Subsequent population-based surveys supported an occupational origin with overall high male-female ratios in high-risk lowlands, but small sex differences within occupational categories, and low prevalence in non-workers. CKDnt was reported in sugarcane and other high-intensity agriculture, and in non-agricultural occupations with heavy manual labor in hot environments, but not among subsistence farmers. Recent studies with stronger designs have shown cross-shift changes in kidney function and hydration biomarkers and cross-harvest kidney function declines related to heat and workload. The implementation of a water-rest-shade intervention midharvest in El Salvador appeared to halt declining kidney function among cane cutters. In Nicaragua a water-rest-shade program appeared sufficient to prevent kidney damage among cane workers with low-moderate workload but not among cutters with heaviest workload. Studies on pesticides and infectious risk factors have been largely negative. Non-occupational risk factors do not explain the observed epidemiologic patterns. In conclusion, work is the main driver of the CKDnt epidemic in Mesoamerica, with occupational heat stress being the single uniting factor shown to lead to kidney dysfunction in affected populations. Sugarcane cutters with extreme heat stress could be viewed as a sentinel occupational population. Occupational heat stress prevention is critical, even more so in view of climate change.

## RESUMEN

La mortalidad por la epidemia de enfermedad renal crónica de origen no tradicional (ERCnt) en Mesoamérica asciende a decenas de miles de personas, principalmente hombres jóvenes. No existe consenso sobre su etiología. En la década de 1990, informes anecdóticos apuntaban como factor de riesgo al trabajo en plantaciones de caña de azúcar; se consideró como posibles causantes a los plaguicidas y el estrés térmico. Estudios de prevalencia de base poblacional subsiguientes apoyaron un origen ocupacional, con una proporción elevada de hombres respecto de las mujeres en las tierras bajas donde el riesgo era elevado, pero con pequeñas diferencias de sexo dentro de las categorías ocupacionales y baja prevalencia en el ámbito no laboral. Se reportó ERCnt en los trabajadores de la caña de azúcar y otros cultivos con alta exigencia física y en ocupaciones no agrícolas que implican trabajo manual intenso en ambientes calurosos, pero no entre los agricultores de subsistencia. Estudios recientes con diseños más sólidos han demostrado cambios en la función renal y en los biomarcadores de hidratación en el curso de los turnos laborales, y disminución de la función renal relacionada con el calor y la carga de trabajo en el curso de la cosecha. La implementación de una intervención basada en la provisión de agua, descanso y sombra a mitad de la cosecha en El Salvador detuvo la disminución de la función renal en los cortadores de caña. En Nicaragua, un programa de provisión de agua, descanso y sombra evitó la lesión renal en los trabajadores de la caña con una carga de trabajo baja y moderada, pero no entre los cortadores, quienes tienen la mayor carga de trabajo. Los estudios sobre plaguicidas y factores de riesgo infecciosos han sido en gran medida negativos. Los factores de riesgo no ocupacionales no explican los patrones epidemiológicos observados. En conclusión, el trabajo es el principal desencadenante de la epidemia de ERCnt en Mesoamérica, y el estrés térmico ocupacional es el único factor que consistentemente conduce a la disfunción renal en las poblaciones afectadas. Los cortadores que trabajan en los ingenios azucareros y están expuestos a un estrés térmico extremo podrían ser considerados una población ocupacional centinela. La prevención del estrés térmico ocupacional es crítica, más aún si se consideran los efectos del cambio climático.

## RESUMO

A mortalidade devida à epidemia de doença renal crônica de origem não tradicional (DRCnt) na Mesoamérica atinge dezenas de milhares de pessoas, principalmente homens jovens. Não há consenso sobre a sua etiologia. Na década de 1990, informações empíricas apontavam o trabalho em plantações de cana de açúcar como um fator de risco; pesticidas e estresse térmico também eram considerados causas possíveis. As pesquisas populacionais subsequentes sustentam uma origem ocupacional da doença, com uma elevada proporção de homens quando comparados à de mulheres, em areas de baixa altitude, onde o risco da doença é mais elevado, mas com pequenas diferenças de gênero quando se consideram as categorias ocupacionais e baixa prevalência no ambiente não-ocupacional. A DRCnt foi identificada em trabalhadores agrícolas da cana de açúcar e de outras culturas que exigem trabalho físico de alta intensidade e em ocupações não agrícolas envolvendo um trabalho manual intenso em ambientes quentes, mas não entre os agricultores de subsistência. Estudos recentes com desenhos mais robustos demonstraram mudanças na função renal e nos biomarcadores de hidratação ao longo dos turnos de trabalho, e diminuição da função renal relacionada à exposição ao calor e à carga de trabalho ao longo da colheita. A implementação de uma intervenção de água-descanso-sombra durante a colheita em El Salvador interrompeu o declínio da função renal em cortadores de cana. Na Nicarágua, intervenções água-descanso-sombra parecem ter sido suficientes para evitar lesões renais em trabalhadores canavieiros com cargas de trabalho baixa e moderada, mas não entre os cortadores de cana que têm carga de trabalho mais pesada. Estudos sobre exposição a pesticidas e a fatores de risco de origem infecciosa têm sido largamente negativos. Os fatores de risco não-ocupacionais não explicam os padrões epidemiológicos observados. Em conclusão, ocupação é o principal desencadeador da epidemia de DRCnt na Mesoamérica, e o estresse térmico ocupacional é o único fator comprovado que leva à disfunção renal nas populações afetadas. Os cortadores de cana que trabalham em engenhos de açúcar e expostos a um estresse térmico extremo podem ser considerados uma população ocupacional sentinela. A prevenção do estresse térmico ocupacional é crítica, especialmente considerando os efeitos das mudanças climáticas.

## CKDnt IN MESOAMERICA: A DEVASTATING EPIDEMIC

An epidemic of chronic kidney disease (CKD) unrelated to diabetes, hypertension and other known causes devastates the Pacific coasts of Nicaragua, El Salvador, Costa Rica and Guatemala (1). The epidemic emerged, initially unnoticed, in the 1970s (2,3). Tens of thousands of deaths have occurred among young male agricultural workers (3,4), particularly sugarcane cutters (3). The Pan-American Health Organization recommends ‘chronic kidney disease of non-traditional origin (CKDnt)’ as the name of the disease (5). Many etiologic hypotheses have been discussed in international workshops (6-11). In this paper we propose that the existing epidemiologic evidence is sufficient to consider CKDnt in Mesoamerica an occupational disease driven by work-related heat stress that warrants immediate preventive action.

## EARLY ANECDOTAL EVIDENCE

CKDnt was first noted by clinicians in the 1990s. In 1996, the main sugarcane enterprise in Nicaragua started pre-employment screening of serum creatinine (SCr) in response to high CKD morbidity and mortality in the neighboring communities from where it sourced its manual labor. In 2000, the company doctor conducted an unpublished epidemiologic study with >900 workers that compared subjects with high SCr to healthy controls. He concluded that strenuous physical work in high environmental temperatures without proper hydration leads to repeated “heat fatigue syndrome” and subsequently to CKD (12). By the early 2000s, increased mortality rates from end-stage kidney disease (ESKD) were recognized primarily in men in the departments Chinandega, León, Rivas and Granada, Nicaragua, where sugarcane is the dominant crop, further supporting an occupational etiology (6). In El Salvador, nephrology wards started to overflow with ESKD patients, the majority being young, male agricultural workers from Pacific coastal regions without known risk factors (13). Nephrologists in the capital of Costa Rica who received kidney patients from Guanacaste on the Pacific Coast, began to call the entity “sugarcane worker nephropathy” (14). Most clinicians viewed CKDnt as an occupational disease and suspected it was related to pesticides.

## PREVALENCE STUDIES SUGGEST OCCUPATIONAL ETIOLOGY

Though KDIGO (Kidney Disease: Improving Global Outcomes, a global organization developing and implementing evidence based clinical practice guidelines in kidney disease) guidelines state repeated SCr measurements are required to diagnose CKD clinically (15), a single determination of biomarkers of kidney dysfunction is considered sufficient on group level, such as in prevalence studies, to give an accurate indication of disease prevalence (16). Between 2010-2015, population-based surveys in communities with different economies observed high prevalence of reduced kidney function (estimated glomerular filtration rate (eGFR) <60 ml/min/1.73m^2^) in men of agricultural communities in coastal lowlands (range 14-42%) with lower frequencies in women (4-10%) and, recently, 13% of men and 5% of women nationally in El Salvador (Table 1) (17-25). In communities without observed excess risk for kidney dysfunction, sex differences were small or absent as well as, noteworthy, within occupational categories. For example, in two high-risk communities in Nicaragua the overall M:F ratio of 4.0 became 1.3 among agricultural workers (17). Female agricultural workers also appeared at increased risk for reduced kidney function in a large survey in León, Nicaragua (24). In El Salvador, for each ten years of work on coastal sugarcane or cotton plantations the risk for men tripled and more than doubled for women (20). These findings suggested that the higher prevalence among men was more likely due to the preponderance of men in high-risk occupations rather than inherent differences between sexes.

Prevalence studies have added information about occupations at risk for CKDnt, both agricultural and non-agricultural. While it is often stated that young, male agricultural workers are disproportionally affected by CKDnt (18,19,23,24,26,27), not all types of agricultural workers are affected equally, and some non-agricultural occupations also carry increased risks. In agriculture, the highest risk for CKDnt is for sugarcane cutters and field workers (17,20,22,28) with CKDnt also reported for cotton, banana, rice and corn workers (17,19,20,23,29). These are industrial crops and contrast with findings from El Salvador (20) and Nicaragua (17,21,30) where kidney dysfunction was not observed among subsistence farmers in coastal areas and coffee farmers who had never worked in plantation agriculture. It was suggested that small farmers have more control over their working conditions than laborers (31). Non-agricultural occupations with reported moderate-high prevalence of kidney dysfunction are construction workers 5-15%, miners 6-16%; port workers with heavy labor (8%), fishermen 7%, and shrimp farm workers 10% (17,28,30,32). A recent case-control study in a Nicaraguan mining area found an >4-fold risk for low kidney function among those who had ever worked in mining or construction (33). Of note, most prevalence studies analyzed current occupation, which may overlook risks from past occupations or falsely identify a current occupation as at high risk. Yet, current occupations with high prevalence of low kidney function are characterized by intensive physical workload outdoors in high environmental heat.

Heat stress, resulting from a combination of metabolic and external heat, has frequently emerged from prevalence studies as a major risk factor for CKDnt. In Nicaragua, lifetime hours/days of cutting sugarcane was strongly associated with eGFR <60 ml/min/1.73m^2^, and the risk for ever cutting cane during the hot dry season was 4-fold, but CKD was not related to lifetime days in less physically demanding jobs (22). Furthermore, a cross-sectional study comparing active workers in different occupations showed that the general health of cane cutters was significantly better than that of construction workers and subsistence farmers who had never worked in sugarcane, whereas markers of kidney health were significantly worse, and low kidney function was associated with years of work in sugarcane (30). In contrast, prevalence studies failed to identify clear associations between pesticides and kidney dysfunction (18,19,22,23,29).

**TABLE 1.** TABLE 1. Male and female prevalence of low kidney function in communities in relation to main economic activities and altitude, and findings for occupational and non-occupational risk factors in prevalence surveys

Country (Reference) (Age of study population)	Type of community (Altitude) (N males, N females)	Population prevalence for M and F (eGFR < 60 ml/min/1.73 m^2^)	M:F ratio at community level	Findings for occupational risk factors	Findings for non-occupational risk factors
**Nicaragua**					
Torres et al, 2010 (17) (20-60 yrs)	All 5 communities (M 479, F 266)	M 14.2% F 3.2%	3.1	Male and female kidney dysfunction within occupational categories:	Pos: hypertension (men), age, NSAIDs (women, not significant), low altitude (except service village at sea level)
	2 high risk communities (sugarcane / mining) (100-300 masl) (M 231, F 266)	M 17.9% F 4.5%	4.0	Agricultural workers (M 60/236, F 4/21): M:F ratio 1.3	Neg: diabetes, hypertension (women), urinary tract infection, obesity, NSAIDs (men)
	Fishing community (sea level) (M 76, F 90)	M 10.5% F 2.2%	5.7	Fishery (M 4/48, F 0/4): M 8.3% vs F 0%	
	Coastal service community (sea level) (M 50, F 90)	M 0% F 0%	-	Services (5/74, 4/83): M:F ratio 1.4	
	Coffee higher altitude (700-1300 masl) (M 40, F 37)	M 7.5% F 0%	-	Economically inactive population: Students (M 0/7, F 0/8): M 0%; F 0%	
Sanoff et al, 2010 (29)(≥ 18 yrs)	Volunteer study in 9 municipalities of León-Chinandega (0-300 masl) (M 848, F 149)	M 14.0% F 3.4%	4.1	Homeworkers, all F (20/472): 4% Increased risk for reduced kidney function: agricultural field work, sugar mill work, rice, corn and banana. Not associated with reduced kidney function: agricultural non-field work, sesame seed, cotton, beans, other crops, cattle Work with pesticides weakly associated with reduced kidney function	Pos: age, family history CKD, illegal alcohol, high water intake Neg: diabetes, hypertension, alcohol, high BMI protective
O'Donnell et al, 2011 (18) (18-56 yrs)	Rural, non-specified agriculture (under and over 500 masl) (M 298, F 473)	M 20.1% F 8%	3.1	Heavy exertion ≥3 hrs: negative Pesticides: Any pesticide use positive (not significant) Mixing or applying pesticides negative	Pos: hypertension, age, low altitude, alcohol (non-significant). Neg: diabetes, obesity, NSAIDs, antibiotic use, illegal alcohol, smoking, water intake, water source
Laux et al, 2012 (21) (20-60 yrs)	Coffee (high altitude) (M 120, F 147)	M 0% F 1.4%	-	93% of men worked with pesticides	Pos: age Neg: diabetes, hypertension, alcohol, NSAIDs
Raines et al, 2014 (22) (15-69 yrs)	Sugarcane CKDu hotspot (sea level) (M 166, F 258)	M 41.9% F 9.8%	4.3	Positive association with years of sugarcane cutting and ever cutting sugarcane in dry season Pesticides: negative for lifetime days mixing/applying; positive for history of accidentally inhaling pesticides (exposure variable not interpretable)	Pos: hypertension, age, alcohol Neg: diabetes, NSAIDs, nephrotoxic medications, smoking, fructose, water consumption
Lebov et al, 2015 (24) (18-70 yrs)	León, urban and rural (low altitude) (M 1054, F 1434)	M 13.8% F 5.8%	2.4	Association with years of agricultural work for both men and women	Pos: diabetes (women), age, hypertension, illegal alcohol, high water consumption Neg: diabetes (men), smoking
**El Salvador**					
Orantes et al, 2011 (19) (≥ 18 yrs)	Bajo Lempa (low altitude) (M 343, F 432)	M 16.9% F 4.1	4.1%	Contact to pesticides/agrochemicals negative	Pos: hypertension, age, family history CKD Neg: diabetes, NSAIDs, markers of metabolic disease, alcohol, obesity
Peraza et al, 2012 (20) (20-60 yrs)	All 5 communities (M 256, F 408)	M 6.6% F 2.9%	2.3	For each 10-y coastal plantation work (sugarcane / cotton):	Pos: diabetes (women), hypertension (women), kidney stones (men), age,
	Two coastal sugarcane communities (M 113, F 175)	M 19.6% F 8.0%	2.8	Men OR 3.1 (95% CI 2.0, 5.0); women OR 2.3 (95% CI 1.4, 3.7)	low altitude, ever smoking (men, not significant)
	3 high altitude communities (M 143, F 233)	M 0.7% F 1.3%	0.8	Subsistence farmers without history of plantation work in lowland communities had no increased kidney dysfunction	Neg: diabetes (men), hypertension (men), obesity, NSAIDs, alcohol (legal and illegal)
	Sugarcane (500 masl) (M 56, F 64)	M 1.8% F 3.1%	0.6		
	Coffee (1650 masl) M40, F 84)	M 0% F 0%	-		
	Service (650 masl) (M 47, F 85)	M 0% F 1.2%	-		
Orantes et al, 2014 (23) (≥ 18 yrs)	Two high-risk agricultural communities: Bajo Lempa (low altitude) Guayapa Abajo (100 masl) (M 793, F 1017)	M 18.0% F 4.2%	4.3	Association with agricultural occupation Positive association for contact with methyl parathion and methomyl in Las Brisas (no data provided on kind of exposure, variable not interpretable)	2 agricultural communities: Pos: hypertension, age Neg: diabetes, no other factors reported.
	Suburban: Las Brisas (570 masl) (M 183, F 395)	M 13.1% F 13.4%	1.0		Las Brisas: a pesticide storehouse was dismantled by the community; 9 out of 10 wells showed contamination with toxaphene. Pos: age, family history CKD, smoking Neg: no data provided
Orantes-Navarro et al, 2019 (25) (≥ 18 yrs)	National survey (altitude varying) (M 1706, F 3111)	M 13.2 F 5.0%	2.6	Higher prevalence of CKD and CKDnt in farmers than in nonfarmers	None

Pos, positive finding; neg, negative finding; NSAIDs, nonsteroidal anti-inflammatory drug; masl, meters above sea level; CKDnt, chronic kidney disease of non-traditional origin

Occurrence of abnormal kidney function in *non-working women and children* has been repeatedly presented as evidence for a non-occupational etiology (34,35). As stated above, for CKD stage 3 and higher (eGFR <60 ml/min/1.73m^2^), Nicaraguan and El Salvadoran cross-sectional surveys report 4-10% for female populations in regions with high risk of CKDnt, including economically active and inactive women (17-25). Yet, female CKD mortality in Nicaragua and El Salvador exceed national female CKD mortality compared with neighboring countries (3), and women in the endemic region of Guanacaste, Costa Rica, have double the CKD mortality rate compared to the rest of the country (2). Although these geographically varying mortality data among women have been suggested as possibly related to environmental exposures to toxic agent(s), environmental heat or the same occupational exposures as those of men could be risk factors as well, as in El Salvador, with a dose-response for years of work in sugarcane/cotton not only for men but also for women (20). Additional studies are needed to determine risk factors for CKDnt in affected women.

Studies have reported that children in endemic areas may show early signs of kidney dysfunction, characterized by glomerular hyperfiltration in El Salvador (36), increased biomarkers of tubular injury in adolescents in high risk areas as compared to low risk areas in Nicaragua (37) and even increased incidence of ESKD among children in high risk sugarcane areas in Guatemala (38). Conclusions regarding etiology in children are limited by the possibility of participants avoiding reporting occupational exposures from (illegal) child labor. Other causes of kidney damage could also be explanatory (low birthweight, infectious diseases, malnutrition), leading to increased susceptibility to insults later in life, but such factors have yet to be adequately studied.

## ANALYTICAL EPIDEMIOLOGIC STUDIES HIGHLIGHT OCCUPATIONAL HEAT STRESS AS A KEY RISK FACTOR

Two recent meta-analyses reported insufficient evidence in support of heat stress leading to CKDnt in Mesoamerican studies (39,40). Both suffer the limitations of such reviews given the great heterogeneity of exposure assessment and differences in study duration and outcomes between studies. Here we present a more detailed review, which includes a variety of studies which enhance understanding of the potential role of occupational heat stress as a risk factor for kidney function decline. These studies include short-term cross-shift studies, medium-term cross-harvest cohorts, and a two-year community cohort with a nested case-control (Table 2). A recent PAHO review by CW provides a detailed analysis of these studies including a table summarizing methodological aspects and quality considerations of the studies (41).

Most analytical studies have been conducted among sugarcane field workers, especially burned cane cutters. Cane cutting in industrial agriculture requires unusually high physical exertion, 6-7 days a week over 5-6 months harvest, described as comparable to the first 12 hours of adventure racing and above that of military personnel during multiday operations (42). Thus, substantial metabolic heat load adds to the high environmental heat, resulting in dangerous levels of heat stress (43-44), but studies have only begun to directly measure work effort or estimate core body temperature on large worker populations.

**TABLE 2.** TABLE 2. Analytical epidemiologic studies on CKDnt in Mesoamerica and findings in favor of and against an occupational etiology of CKDnt

Study (Reference) Country	Setting	Findings supporting occupational etiology of CKDnt ^*^	Findings supporting non-occupational etiology of CKDnt
**Cross-shift studies**			
Crowe et al, 2014 (45) Costa Rica	Cane cutters (n=56) 3 sampling days in 1 week, midharvest Urine: dipstick and microscopy	Clear evidence of dehydration and kidney injury among heat stress exposed workers Overnight recovery insufficient	None
García-Trabanino et al, 2015 (46) El Salvador	Cane cutters (n=189) End of harvest Physical exam, urine, blood	Changes for blood pressure and kidney-related serum and urine biomarkers involved in maintaining water and electrolyte balance associated with heat and workload No association with any pesticide use or use of glyphosate, paraquat, 2,4-D, triazines, organo-phosphates, pyrethroids Association with carbamate insecticides	None
Wesseling et al, 2016 (47) Nicaragua	Cane cutters (n= 29), Day1, Day6, Week 9 Administrative referents (n=25), start and end of harvest Physical exam, urine, blood	Dehydration cross-shift among cutters is clear and repeated among cutters and not among administrative workers Largest effects on Day 6, during acclimatization period	None
Wegman et al, 2018 (48) El Salvador	Intervention study Cane cutters (n=40) Pre- and post-intervention (midharvest) and end of harvest	Documented cases of AKI (KDIGO) Cross-shift decline in eGFR related to heat and workload Positive effect of intervention on reducing cross-shift kidney function decline	None
See also Bodin et al, 2016 (56)	Physical exam, urine, blood		
Sorensen et al, 2018 (49) Guatemala	Sugarcane field workers (n=105) 3 monthly days towards end harvest Urine, blood	High prevalence of acute decline in eGFR in well-hydrated workers	Half of study population had HbA1c between 5.7-6.4% and increasing levels predicted cross-harvest AKI
		Increasing temperature, hyperuricemia, decreased urine pH, urinary leukocyte esterase, and serum hyperosmolality were risk factors	
Butler-Dawson et al, 2019 (50) Guatemala	Sugarcane field workers (n=517) 3 monthly days towards end harvest Urine, blood	78% of workers developed cross-shift AKI (KDIGO definition) at least once The majority in well-hydrated workers. Dehydration and insufficient electrolyte intake were risk factors for AKI NSAIDs combined with high pre-shift urinary specific gravity increased risk for AKI	None Since hydration and electrolyte intake, despite being preventive, did not prevent all cases of AKI, the authors concluded that other factors must be contributing to the injury.
**Occupational cohorts**			
Laws et al, 2015 (51) Laws et al, 2016 (52) Nicaragua	Cross-harvest cohort in five job categories of sugarcane field workers and two categories of nonfield workers (n=284)	eGFR decline and increase in markers of acute tubular injury differed by job category Findings in accordance to heat stress in the job categories Protective effect from electrolytes	None
Wesseling et al, 2016 Nicaragua (47)	Cutters (n= 29), Day 1, Day 6, Week 9 of harvest Administrative workers (n=25), cross-harvest	Marked decline in kidney function among cutters but not in the referent population	None
Butler-Dawson et al, 2018 (53) Guatemala	Cross-harvest cohort of cane cutters (n=330)	Decline in eGFR in 37% of young male cutters with 6% with a decline in eGFR > 20% 3% declined over the harvest to eGFR <60 ml/min/1.73m^2^	According to authors, there are possible non-occupational contributory risk factors: overinterpretation of positive findings of association with smoking (low prevalence) and local residence (no further details, may be occupational)
Kupferman et al, 2018 (54) Nicaragua	Retrospective cross-harvest cohort of 326 sugarcane workers (cane cutters, seeders/seed cutters, weeders, pesticide applicators, irrigators) for IKI Prospective follow-up of 34 IKI cases at 6m or 1 yr	10.4% of workers had IKI at end of harvest compared to baseline A third of IKI cases developed CKD over the follow-up period	None
Hansson et al, 2019 (55) Nicaragua	First year cross-harvest assessment of Adelante Initiative Program: observation and measurement of existing water-rest-shade program in a sugarcane company 4 job categories (n=530)	IKI developed more frequently among category of highest physical workload (12-fold risk compared to category of lowest workload) Occurrence of IKI much higher among dropouts (32% vs 7.5%) Current preventive measures insufficient for workers with highest heat stress	None
Gallo-Ruiz et al, 2019 (57) Nicaragua	Brick makers (n= 257) 4-month follow-up	High prevalence of CKD (twice eGFR<60 ml/min/1.73m^2^ ≥ 3 months apart) Risk higher among highest heat exposed (ovens) Low water intake and longest work week associated with decline in eGFR over the follow-up	None
Riefkohl et al, 2017 (68) Nicaragua	Cross-harvest cohort to investigate Leptospira in five categories of sugarcane field workers and two of non-field workers (n=282) Cross-sectional evaluation of non-hired job applicants (n=47) and a sample of miners, construction workers and port workers (n=160)	None Exposure to Leptospira is higher among sugarcane field workers and miners (as expected due to more contact with soil contaminated with rat urine) Some association with biomarkers of kidney injury The study does not provide evidence of Leptospira as a cause of CKDnt.	None
Fischer et al, 2018 (69) Nicaragua	A cohort of incident cases of AKI (n=586) recruited at sugarcane company hospital, to assess predictors for progression to CKD Start of cohort Feb 2015 with case finding till May 2017 (28 months) and follow-up till November 2017 (min 210 days, max 1007 days).	49 (8.5%) AKI patients progressed to CKD, usually within 6 months. AKI occurred more frequently among males and in the job categories with highest internal and external heat exposures.	None
**Intervention studies**			
Wegman et al, 2018 (48) El Salvador See also Bodin et al, 2016 (56)	Cross-harvest with water-rest-shade intervention starting mid-harvest Inland (n=40) with intervention Coastland (n=40) without intervention For cross-shift analyses see above	Cross-harvest percentual decline from pre-harvest baseline values of eGFR occurred in both inland and coastal group but less in the intervention group. After a similar initial downward slope during preintervention, the decline appeared to halt in the inland group after start of the intervention	None
**Community-based studies**			
Gonzalez-Quiroz et al, 2018 (58) Nicaragua	Community cohort with 2-year follow up with five 6-months interval evaluations of healthy community members in high-risk area Age 18-30, including workers and non-workers (n=350; M 263, F 87)	Rapid decline in 10% of healthy men at baseline (n=25) and 3% of women (n=3) Associations with agricultural work, outdoor work, lack of shade, and cane and seed cutting, fever last 6 months No associations with self-perceived heat stress variables	According to authors, there are possible non-occupational contributory risk factors. None was observed or proposed.
Smpokou et al, 2019 (64) Nicaragua	Nested case-control within the community cohort (60): Cases: rapid decliners (n=28) Controls: random sample of healthy participants in the community cohort (n=325, excluding low kidney function at baseline) Comparison of baseline urinary levels of 12 metals, 12 pesticides, and two mycotoxins	Urinary levels of different contaminants varied, most were low or non-detectable, some pesticide levels were considerable. No associations between levels of any contaminant and cases of rapid decline of kidney function. This study did not analyze specifically occupational variables but, conversely, it dismisses an environmental etiology	None
**Other study designs**			
Kupferman et al, 2016 (71) Nicaragua	Cross-sectional study of CKD (n=107) and non-CKD (n=159) members of families affected by CKDnt (at least two members) Aim to gain better insight in distinguishing clinical features of CKDnt in Mesoamerica	Very high serum uric acid levels in family members with CKD as compared to family members without CKD suggest potential causal role in CKDnt. The study could not explore associations with occupation, almost all participants were sugarcane workers	None
Wesseling et al, 2016 (30) Nicaragua	Cross-sectional comparison between cane cutters (n=86), construction workers (n=56), subsistence farmers (n=52) Living and working in the same high-risk area in northwest Nicaragua	Cane cutters with better general health but worse kidney function points to physically demanding work Prevalence of kidney dysfunction indicators highest among cane cutters, intermediate in construction workers and subsistence farmers not affected Hyperuricemia strongly associated with reduced kidney function Negative for any pesticide use and specifically for glyphosate, paraquat, 2,4-D, chlorpyrifos, cypermethrin Electrolyte solution was preventive among cane cutters Association with cumulative time in job among cane cutters	Hypertension, age, and high water intake associated with reduced kidney function Alcohol, smoking and obesity were negative.
Yih et al, 2019 (33) Nicaragua	Prevalent case-control study on infectious causes in mining area in León Department Cases n=112, controls =176	A 4.4-fold risk of CKDnt for ever work in mining or construction 26% of participants seropositive for at least one strain of Leptospira No associations of Leptospira and Hantavirus seropositivity with CKDnt	None

* For detailed descriptions of studies, see Wesseling C. Evidence for CKDnt being primarily an occupation driven disease in Mesoamerica. Report commissioned by PAHO; 2019 (41). CKDnt, chronic kidney disease of non-traditional origin; AKI, acute kidney injury; IKI, incident kidney injury; NSAID, nonsteroidal anti-inflammatory drug

*Cross-shift* evaluations have been conducted in sugarcane recurrent dehydration from strenuous work in hot environ-cutters in Costa Rica, Nicaragua, El Salvador and Guatemala. ments (45-50). Three studies examining multiple cross-shifts Without exception, these studies concluded that cross-shift documented repeated dehydration over the day (45,47,48). In physiologic and biomarker changes were compatible with one, 27% of cane-cutters started already with high urine specific gravity (≥1025) in at least one out of three observed shifts and 52% had high urine specific gravity at least once at the end of the shifts, indicating that dehydration occurred over the workday and that overnight recovery was insufficient (45). The start of the harvest, when workers acclimatize to the physically demanding work, was associated with the largest cross-shift differences in hydration and kidney function (47). In a waterrest-shade intervention study in El Salvador, the cross-shift decline in eGFR related to workload (tons cut); moreover, the intervention appeared to reduce cross-shift kidney function decline (48). In Guatemala, an ostensibly intense hydration regimen was implemented, but indicators of poor hydration were still associated with kidney function decline and biomarkers of kidney injury during the workday (49). Among these cane cutters, increases in SCr compatible with the KDIGO acute kidney injury definition occurred in 78% at least once during three measurement days (50). Other studies have also reported changes similar in magnitude during the workday (47,48).

Cross-harvest studies in Nicaragua, El Salvador and Guatemala found, without exception, decline in eGFR among field job categories, more severe in workers with the highest combination of environmental and metabolic heat exposures (47,48,51,55). A study in Nicaragua observed larger decline in kidney function among fieldworkers as compared to factory workers and drivers, consistent with degree of heat stress (51). Irrigators had a larger eGFR decline than cutters despite lesser physical demands. As dropouts were not followed up it is likely this represents a healthy worker effect among cutters as was demonstrated years later in the same company (55). Laws et al. also observed an increase over the harvest in markers of acute kidney injury (NGAL, NAG and IL-18), highest among cane cutters with the highest physical workload (52). Another study in Nicaragua documented a cross-harvest decline in eGFR of 10 ml/min/1.73m^2^ among cane cutters in only nine weeks, but not in administrative workers. In this study, cross-shift changes at the start of the harvest predicted the longitudinal kidney function decline (47). In Guatemala, 6% of young male cutters and other field workers had a cross-harvest eGFR decline >20% of baseline (53). In a retrospective cohort analysis, 19% of cane cutters, who have the highest physical workload, developed kidney injury at end of harvest, compared to less than 6% of other field workers, and no kidney injury among pesticide applicators or irrigation workers (54).

In Nicaragua, sugarcane workers with low-moderate physical demand maintained kidney function over the sugarcane harvest, but for those with heavy and very heavy physical workload (seed and burned cane cutters, respectively) the workplace practices in place for providing water-rest-shade appeared insufficient to prevent kidney function decline (cross-harvest change in eGFR -5 and -9 ml/min/1.73m^2^, respectively). Cases of cross-harvest incident kidney function injury, compatible with KDIGO acute kidney injury criteria were 2% in the lowest physical workload category versus 27% in burned cane cutters, a 12-fold excess (55).

A water-rest-shade intervention was introduced mid-harvest in one of two work teams at a sugar mill in El Salvador. After similar initial eGFR decline (measured in % change from baseline) for both work groups over the first two months of the harvest, the kidney function stabilized in the intervention group, but not in the non-intervention group (48, 56). A limitation of the study was that the intervention was not in the hottest area, but a previous cross-shift study (46) showed that cane cutters in the highland intervention area also have reduced eGFR.

One non-agricultural cohort of brickmakers found 13% with CKD (twice eGFR <60 ml/min/1.73m^2^, 4 months apart). Workers with the highest heat exposure were most affected. Low water intake and longer work week was associated with higher degree of decline in eGFR (57).

A community-based cohort study in a high-risk area in Nicaragua, following 350 adults under age 30, found that 9.5% (n=25) of the male and 3.4% (n=3) of the female population showed a pattern of rapid decline in kidney function over the course of two years (58). Rapid decline was associated with outdoor work, lack of shade, and work in agriculture and sugarcane, but not with years of employment in sugarcane or self-perceived heat stress and heavy workload. The authors concluded therefore that also non-occupational factors must be influencing disease occurrence, but no such factors were identified or proposed.

Further support for occupational heat stress as a key factor in the etiology of CKDnt in Mesoamerica is provided by experimental animal data. Recurrent dehydration from heat exposure caused tubulointerstitial kidney injury in mice deprived of water during daily heat exposure, despite drinking the same total amount of water during the night as mice with free access to water over 24 hours (59). A recent study showed that increasing the core temperature in heat exposed mice led to kidney injury with a similar histopathological appearance as initial kidney injury among sugarcane workers, consistent with hyperthermia playing a role (60). In a quasi-experimental study in marathon runners, 55% developed acute kidney injury, specifically tubular damage associated with salt and water loss due to sweating (61). An extensive overview of the pathophysiology of acute and chronic kidney damage from physical work in the heat has been recently published (62).

## SCARCE EVIDENCE FOR OTHER OCCUPATIONAL OR ENVIRONMENTAL RISK FACTORS

### Pesticides and metals

Exposures to pesticides continue to be a concern as a potential risk factor for CKDnt in Mesoamerica. A comprehensive review from 2017 (63) concluded that there was scarce evidence for an association, but recommended studies with adequate exposure assessment before definitely discarding pesticides as an important risk. More recently, the above-mentioned Nicaraguan community cohort did not observe an association of self-reported pesticide use and kidney dysfunction, including glyphosate, paraquat, cypermethrin and methomyl (58), and a case-control study nested in this cohort found no association between rapid decline of eGFR over two years and baseline urinary levels of 12 common pesticides or their metabolites, including 2,4-D, glyphosate, chlorpyrifos and pyrethroid insecticides (64). To date, the only suggestive finding in Mesoamerica in relation to pesticides is a 13% prevalence of CKD stage ≥3 in Las Brisas, El Salvador, similar for men and women, attributed to local groundwater contamination with toxaphene after the dismantling of a pesticide storehouse by the community (23), which suggests an isolated situation. One of the two above mentioned meta-analysis studies reported an association between pesticide use and CKDnt (OR 1.35, 95%CI 0.98, 1.87) based on 13 studies (7 from Central America), but the meta-analysis had similar problems as with the assessment of heat stress (40).

Exposures to metals and metalloids have been considered as risk factors for CKDnt in Mesoamerica, especially arsenic as it has been detected at varying levels in water and soil in this volcanic region (65). In Nicaragua, lead, cadmium, and uranium were not associated with biomarkers of kidney injury or kidney function among sugarcane workers and miners, whereas total arsenic was associated with kidney injury among workers with the highest urinary levels (28). However, a longitudinal study following urinary levels of 12 metals in the Nicaraguan general population found no association with cases of rapid kidney function decline (64). A systematic assessment of the probability that metals were potentially important, concluded that this was highly unlikely, firstly because no particularly high exposures have been found in populations at risk as compared to established dose-response relationships, secondly because the clinical and histopathological presentation of CKDnt is not in accordance with the usual renal metal toxicity findings (66).

Potential nephrotoxic effects from toxic exposures may be magnified by high physical workload or by genetic predisposition. Several of the above-mentioned cross-shift and cross-harvest studies on heat stress simultaneously addressed pesticide exposures. Except for an isolated association between low pre-shift eGFR and self-reported use of carbamate insecticides in El Salvador (46,63), none of these studies found associations with pesticide use; interactions were not examined (48,51,53,55,58,63). However, the relative difficulty of measuring environmental exposures to toxicants (such as pesticides) or heat stress could obscure their potential role, when compared to potential genetic determinants which typically have less measurement error (67).

Even so, for a toxicant to be a key cause of an epidemic of the magnitude seen in Mesoamerica, it must be present during prolonged time periods in multiple countries, and be widespread in occupational or environmental settings in high-risk areas but not in low-risk areas. A pesticide, metal or other toxicant with such characteristics has not been identified. Toxic agents and their potential role as disease initiators interacting with heat stress or other putative exposures, or as disease progressors need to be addressed, but a main role of toxicants in the etiology of this epidemic is considered unlikely.

### Infectious agents

Interest in an infectious etiology has been increasing, particularly the endemic vector-borne diseases leptospirosis, dengue, zika, chikungunya and malaria. All could have an occupational component, all could potentially exacerbate effects of heat stress, and all could affect disease progression, but few data exist so far. Based on observations of acute nephropathy in sugarcane workers and infestation of rodents in sugarcane fields in Nicaragua, leptospirosis or Hantavirus were hypothesized initially (32). Leptospira seropositivity was highest among sugarcane cutters as compared to other job categories in cane production and seropositive workers had higher NGAL levels, but there were no associations with kidney dysfunction that could explain the CKDnt epidemic (68). A recent prevalent case-control study in a mining area of Nicaragua observed a high rate of Leptospira seropositivity (26%) among study participants but did not find a positive association with CKDnt; Hantavirus seropositivity was not associated either (33). The observed acute early phase of the disease points to a systemic inflammatory process, which was hypothesized to be due to an exogenous infectious or toxic cause (69). However, sugarcane workers attending the company hospital with acute kidney disease did not have raised titers of leptospirosis or Hantavirus, and probing for pathogen genomes using advanced molecular methods in blood, urine and kidney tissue in a subset (n=10) did not reveal any DNA or RNA patterns suggesting involvement of any known or novel pathogen (70). In a recent cross-harvest cohort, the inflammatory marker C-reactive protein increased in association with incident kidney injury, which may reflect heat-induced inflammation (55). A possible interaction between heat stress and infections of any kind –i.e., increased kidney stress while working hard in heat with an ongoing infection– has not yet been explored but should be of priority.

## NON-OCCUPATIONAL RISK FACTORS

Many non-occupational factors have been hypothesized to explain the CKDnt etiology and findings are represented in Tables 1 and 2. For a non-occupational risk factor to be explanatory, it should be able to explain the observed occupational epidemiological patterns of CKDnt. CKDnt by definition excludes diabetes and hypertension but, in any case, diabetes prevalence is very low in affected areas and hypertension may also be a consequence of kidney disease and is therefore ambiguous as a risk factor. Urinary infections, kidney stones, obesity, and biomarkers of metabolic disease have been infrequently associated with kidney dysfunction. Hyperuricemia has been associated with kidney dysfunction in cross-sectional studies (30,46,47,71), tentatively as part of the pathophysiological pathway compatible with an occupational heat stress etiology (72). Consumption of NSAIDs, which also can be seen as occupationally related if used because of musculoskeletal pain in hard manual labor, yielded negative results in all studies, except for an interaction with pre-shift dehydrated state for acute kidney injury in a Guatemalan cross-shift study in sugarcane field workers (50), and there are no consistent observations for other nephrotoxic pharmaceuticals. Alcohol, illegal alcohol and tobacco were inconsistently related to low kidney function in prevalence studies. Smoking was considered a risk factor in a cross-harvest study in sugarcane workers in Guatemala (OR of 5.27) (53), but the prevalence of ever smoking was only 10%.

Genetic susceptibility may also be an important trigger for clusters of CKDnt (73). One study in a hotspot in Nicaragua hypothesized that the much higher levels of uric acid in CKDnt patients as compared to family members without CKDnt suggests an extreme phenotype on the disease spectrum (71). Genetic susceptibility has not been explored, but future studies could shed light on disease mechanisms.

## TOWARDS UNDERSTANDING THE NATURAL HISTORY OF CKDnt

Recently, studies on the progression of acute kidney injury and incident kidney injury have been undertaken. One study examined 34 workers with cross-harvest incident kidney injury 6 and 12 months later; 10 (34.5%) progressed to eGFR <60 ml/min/1.73m^2^ and 11 (38%) to a decline of eGFR >30% of baseline (54). After proposing that CKDnt starts with an acute adverse kidney event (32), sugarcane workers with acute kidney injury episodes were captured at the company hospital in Nicaragua and then followed up for progress to CKD (69). Acute kidney injury occurred more frequently among male workers and in cutters and other fieldworkers. Of the 586 acute kidney injury patients, 49 (8.4%) progressed to CKD, most within six months.

Most analytical epidemiology studies are informative about short- or medium-term damage but only few on incidence of true CKDnt, i.e. a disease state which is not reversible. Functional changes in the kidneys during and after physically hard work in heat is also explored in sports medicine, clearly showing considerable short-term changes compatible with acute kidney injury (61,62,74-76). In occupational settings the heat stress is repeated day after day, a situation that cannot be evaluated in strictly controlled experimental settings. Workers’ hydration status has been considered as essential on the pathophysiological pathway to kidney disease (62,77,78), but it is gradually recognized that preventing dehydration may not be enough to protect workers from increasing core body temperature from exertional heat in hot environments (79,80) and consequent adverse effects on the kidneys (55). This understanding will have impact on the design of occupational interventions. There is an obvious need for longer-term longitudinal occupational studies, with minimal loss of follow-up.

## CONCLUSIONS

Studies have documented that CDKnt in Mesoamerica is an occupational disease associated with heavy manual work in hot, often agricultural, regions along the Pacific coast. Importantly, no study has provided evidence against an occupational nature of CKDnt (41). Of all potential risk factors, heat stress appears to be the strongest, although much is still needed to better understand determinants of CKDnt onset. Improved exposure assessment and longer follow-up will be important in understanding disease progression. Early research shows benefit of water-rest-shade programs to prevent kidney dysfunction. To date no specific occupational or environmental toxicants have been identified that can adequately explain the epidemic. Non-occupational risk factors have not been consistently associated with CKDnt, and no factor emerged suspect as a primary driver. The evidence for occupational heat stress is sufficiently strong that it requires primary prevention, with evaluation of the components and effectiveness of implementation of the interventions. Future studies should also focus on disease initiation, progression and secondary prevention.

In summary, we consider the evidence sufficient that work is the main driver of the epidemic of CKDnt in Mesoamerica. Occupational heat stress is the single factor shown to lead to kidney dysfunction in affected populations. Sugarcane cutters could be viewed as a sentinel occupational population. With increasing environmental temperatures, risk may extend to other occupations with lesser physical demands and other regions as they warm. Action must be taken now while we continue to refine understanding.

### Authors’ contributions.

David Wegman, Kristina Jakobsson, Christer Hogstedt, Richard Johnson, Rebekah Lucas, Erik Hansson and Sandra Peraza are part of the “La Isla Network International Research Group”. This work has been possible thanks to the collective efforts of our research group affiliated to La Isla Network and the support of other researchers. Design: CW, JG, JR-G, DHW and KJ; Acquisition of data: CW and contributions of all authors; Interpretation: all authors; Drafting: CW, JG, DHW, KJ; Revising critically for intellectual content: all authors; Final approval of submitted version: all authors; Agreement to be accountable for all aspects of the work: all authors.

### Funding.

The Pan-American Health Organization provided funding for the preparation of an earlier technical report, on which this article is based.

### Disclaimer.

Julietta Rodríguez-Guzman and Agnes Soares da Silva are staff at the Pan American Health Organization. Authors hold sole responsibility for the views expressed in the manuscript, which may not necessarily reflect the opinion or policy of the RPSP/PAJPH or the Pan American Health Organization (PAHO).
